# Mapping Remote Subcortical Ramifications of Injury after Ischemic Strokes

**DOI:** 10.1155/2014/215380

**Published:** 2014-04-24

**Authors:** Leonardo Bonilha, Travis Nesland, Chris Rorden, Paul Fillmore, Ruwan P. Ratnayake, Julius Fridriksson

**Affiliations:** ^1^Department of Neurology, Medical University of South Carolina, Charleston, SC 29425, USA; ^2^Comprehensive Epilepsy Center, Division of Neurology, Medical University of South Carolina, Charleston, SC 29425, USA; ^3^Department of Psychology, University of South Carolina, Columbia, SC 29208, USA; ^4^Department of Communication Sciences and Disorders, University of South Carolina, Columbia, SC 29208, USA

## Abstract

*Background*. The extent of brain damage in chronic stroke patients is traditionally defined as the necrotic tissue observed on magnetic resonance image (MRI). However, patients often exhibit symptoms suggesting that functional impairment may affect areas beyond the cortical necrotic lesion, for example, when cortical symptoms ensue after subcortical damage. This observation suggests that disconnection or diaschisis can lead to remote cortical dysfunction that can be functionally equivalent to direct cortical lesions. *Objective*. To directly measure subcortical disconnection after stroke. *Methods*. We describe a principled approach utilizing the whole brain connectome reconstructed from diffusion MRI to evaluate the reduction of apparent white matter fiber density in the hemisphere affected by the stroke compared with the spared hemisphere. *Results*. In eight chronic stroke patients, we observed subcortical disconnection extending beyond the location of tissue necrosis and affecting major white matter pathways underlying the necrotic area. *Conclusions*. We suggest that it is possible to detect and quantify previously unappreciated areas of subcortical and cortical disconnection. Specifically, this method can be used to evaluate the relationship between lesion location and symptoms, with emphasis on a connectivity-based approach.

## 1. Introduction

Hypoxic damage resulting from an ischemic stroke may lead to liquefactive necrosis and permanent damage of hypoperfused brain structures [1]. Several factors play a role in determining the final location and extent of structural brain damage, but the degrees of decreased blood flow are crucial determinants of irrevocable cell death and breakdown of tissue architecture [2].

The full extent of the structural compromise resulting from an ischemic stroke is usually appreciated after weeks or months have elapsed since the acute stroke, when most of the necrotic process composed of inflammation, gliosis, and reabsorption of damaged cells has occurred. In routine clinical practice, this is assessed in the chronic stage by high-resolution magnetic resonance imaging (MRI) studies, specifically through T1 and T2 relaxation based sequences [3].

Even though the location of poststroke necrotic tissue can be accurately defined based on these MRI sequences, it is often observed that patients may exhibit neurological impairments that are beyond the expected deficits from the magnitude of the stroke as defined by clinical MRI [4, 5]. For example, severe aphasia can be observed in patients with relatively small lesions in the left hemisphere [4]. Likewise, other typical cortical dysfunction signs such as spatial neglect or cortical sensory loss can be observed in patients with strictly subcortical strokes [6]. This phenomenon is often attributed to functional disconnection or diaschisis [7], suggesting that the dysfunctional region is not directly damaged by the stroke, but it is indirectly affected by the loss of connections resulting from the stroke [8].

It is an important conclusion from this phenomenon that we often fail to appreciate the full extent of structural abnormalities resulting from a stroke by only evaluating tissue necrosis using routine clinical MRI. Loss of connectivity may lead to impairments that are functionally equivalent to direct tissue necrosis.

In this study, we aimed to assess the full extent of white matter disconnections in chronic stroke patients. We used diffusion MRI to assess the extent of white matter injury observed in chronic stroke. We hypothesized that the location of structural abnormalities due to stroke can extend far beyond the location of tissue necrosis identified on clinical MRI.

## 2. Methods

### 2.1. Subjects

We studied eight patients (mean age 52 ± 7.2 years) who suffered a left hemisphere ischemic stroke at least six months prior to enrolling in this study. The demographic information from all patients is summarized in Table 1. All patients were right handed. They did not have a history or imaging evidence of other previous strokes and had no history of other neurological illnesses. All patients signed an informed consent to participate in this study. The Institutional Review Board at the University of South Carolina approved this study.

### 2.2. Behavioral Measures

All patients underwent a comprehensive assessment of language performance employing the Western Aphasia Battery [9] and the Boston Naming Test [10], yielding a global measure of aphasia (the aphasia quotient) and scores related to speech fluency, comprehension, repetition, content, and object naming. These data are summarized in Table 1.

### 2.3. Image Acquisition

All subjects underwent MRI scanning at a 3T Siemens Trio equipped with a 12-channel head coil located at the University of South Carolina, yielding (1) T1-weighted images (3D MP-RAGE, TR = 2250 ms, TE = 4.15 ms, 256 × 256 matrix, 256 × 256 mm FOV, parallel imaging GRAPPA = 2, 80 reference lines, TA = 377 s) and (2) diffusion EPI scan (30 directions with *b* = 1000 s/mm^2^, TR = 6100 ms, TE = 101 ms, 82 × 82 matrix, 222 × 222 mm FOV, parallel imaging GRAPPA = 2, 80 45 contiguous 2.7 mm axial slices, TA = 390 s).

### 2.4. Image Processing

MR images were converted to NIfTI format utilizing the dcm2nii tool from the MRIcron software package [11]. The location of the stroke lesion was manually delineated on the T1 image. T1-weighted and diffusion images were normalized into standard MNI space utilizing unified segmentation-normalization routines of the clinical toolbox for the software SPM8 [12]. This process applied cost-function masking based on the lesion mask. In addition to normalizing the T1 and lesion maps, the segmentation provided probabilistic maps of gray and white matter in standard space. The intersection between the probabilistic gray matter map and a Brodmann areas template (distributed with MRIcron) was used to provide cortical regions for the subsequent analyses of the DTI data, where cortical region corresponded to the intersection between a Brodmann area and voxels with greater than 50% of probability of brain gray matter. This step ensured that voxels in the Brodmann area atlas outside the cortical regions were not included as corresponding to gray matter. The DTI volumes were aligned to the B0 image using the FSL FLIRT tool [13]. In diffusion space, whole brain deterministic fiber tractography was reconstructed with the software Diffusion Toolkit (Ruopeng Wang, Van J. Wedeen, TrackVis.org, Martinos Center for Biomedical Imaging, Massachusetts General Hospital) (we applied software default parameters for tractography, including angle threshold = 45 degrees, inclusion mask derived from the average of diffusion weighted signal). Importantly, white matter tractography was reconstructed in the whole brain, in accordance with previously described brain connectome methodology [14–16], irrespective of the origin and termination of fibers. Hence, the extralesional component of fibers that were partially interrupted by the necrotic lesion was still included in the tractography and in the computation of the connectome.

In order to avoid erroneous tracking of fibers in the necrotic lesion due to random diffusion in the stroke cavity, we excluded the stroke lesion from the tractography inclusion mask (i.e., where fiber tractography was calculated).

Streamlines were then transformed back to standard “MNI” space using Diffusion Toolkit. For each fiber, the cortical location of the fiber end points was computed. The number of tractography streamlines connecting each possible pair of Brodmann areas was computed generating the brain connectome, that is, a connectivity matrix for each subject.

### 2.5. Fiber Loss Quantification

From each patient, the connectivity data from the left (lesioned) hemisphere were compared with the homologous connectivity data from the right hemisphere. For each possible “link” between two Brodmann areas in the left hemisphere (Lij), the number of streamlines connecting these two areas was compared with the number of streamlines connections connecting the two homologous areas within the right hemisphere (Ri′j′). Inter-hemispheric links were not computed.

For each link Lij, if Ri′j′ was composed of at least 20 connecting streamlines and if Lij ≤ 0.025 (Ri′j′) (i.e., if the lesioned hemisphere had a reduction in the number of fibers of at least 97.5% compared with nonstroked hemisphere), Lij was considered substantially reduced or damaged.

**Figure 1:**
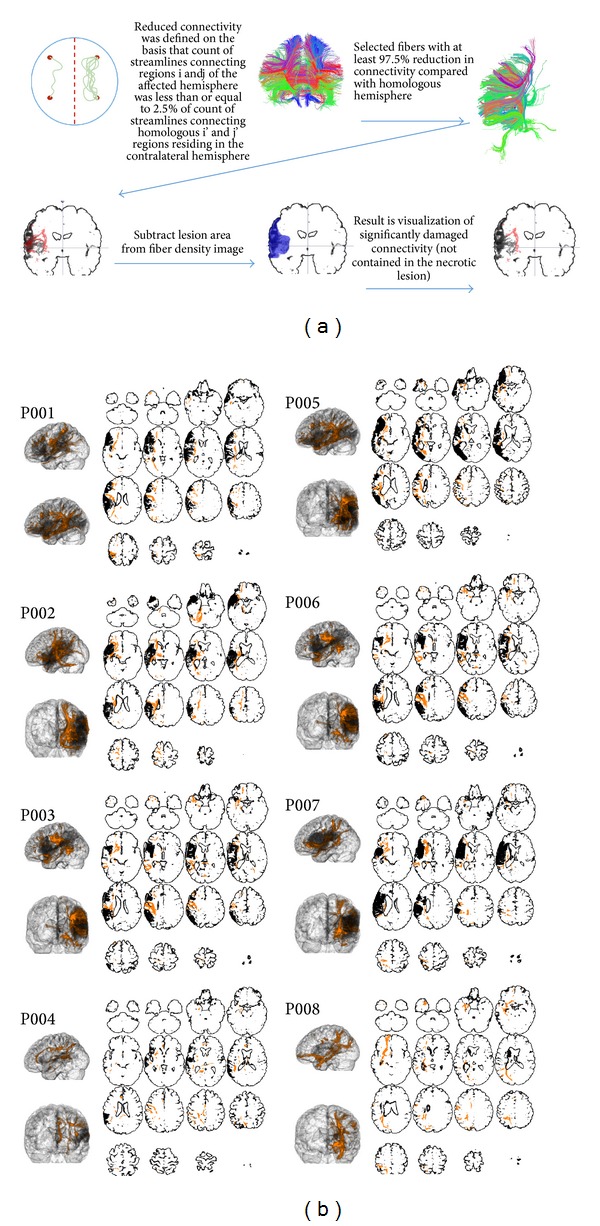
(a) Image processing steps utilized to quantify hemisphere connectivity. A white matter link was considered significantly reduced if the number of fibers connecting a pair of Brodmann areas in the left hemisphere (with stroke) was less than 2.5% of the number connecting the pair of homologous Brodmann areas in the right hemisphere. Fibers located within the necrotic lesion were excluded from this analysis. (b) The original necrotic lesion site (dark gray) and the significantly reduced white matter pathways (orange) relative to the right hemisphere are demonstrated in three-dimensional reconstructions and representative slices for each patient. The images are skull stripped for better visualization of the location of the necrotic tissue.

Specifically, the threshold of 20 streamlines was defined since deterministic tractography may generate sparse connectomes; that is, some pairs of regions have no connecting tracks [16]. Hence, pairs of cortical regions that are inconsistently tracked may appear highly asymmetrical if, for example, 2 streamlines are tracked in one side and no fibers tracked in the other side. In order to avoid erroneous quantification of fiber asymmetry in regions inconsistently tracked, we only evaluated links that contained at least 20 streamlines (i.e., links that were consistently tracked). Similarly, the threshold of 97.5% was arbitrarily chosen to represent a substantial reduction in the number of fibers. These steps are summarized in Figure 1(a).

We also assessed the average number of lesioned fibers in anatomically defined white matter tracks (according to the white matter atlas from Johns Hopkins University (JHU) [17]).

## 3. Results

For each patient, anatomical connections with a high degree of fiber loss (beyond 97.5% compared with the nonstroked hemisphere) were displayed in a three-dimensional diagram of the skull stripped T1-weighted image (Figure 1(b)). This step demonstrates the relationship between the original necrotic area and the anatomical distribution of reduced perilesional connectivity.

All patients exhibited a clear degree of anatomical disconnection not only involving areas immediately adjacent to the necrotic area but also at times encompassing remote locations such as the medial temporal region, occipital lobe, basal nuclei, and the brain stem. While most patients demonstrated a necrotic lesion involving the dorsolateral aspect of the left hemisphere, areas of disconnection extended into distant regions in the temporal, frontal, and occipital lobes.

Even though major white matter tracts were affected in most patients, the location of fiber loss was variable and unique to each patient.

The average voxel-wise fiber reduction per white matter pathway is demonstrated in Table 1. Note that fiber reduction encompassed major white matter pathways in almost all subjects. In an exploratory analysis, employing a threshold of statistical significance of *P* < 0.005 due to the number of multiple comparisons, we observed a significant negative correlation between repetition performance and the number of lesioned fibers in the white matter track termed left cingulum (hippocampus) from the JHU atlas, which represents white matter fibers travelling longitudinally in the temporal lobe [18].

## 4. Discussion

In this study, we aimed to identify the full extent of anatomical reorganization resulting from a previous ischemic stroke. We employed modern imaging techniques to disclose abnormalities in white matter integrity that cannot be appreciated by visual inspection of clinical images, and we demonstrated that significant reduction in intrahemispheric connectivity extends beyond the limits of the necrotic tissue.

The purpose of this paper was to define the anatomical distribution of “invisible” white matter lesion, that is, subcortical ramifications of the ischemic stroke. This study was not designed to directly assess the relationship between regional white matter fiber reduction and behavioral performance due to its limited sample size. Nonetheless, by evaluating the average fiber reduction in anatomically defined major white matter pathways (in accordance with the JHU white matter atlas [17]), we observed that several major white matter pathways exhibited a degree of fiber reduction in most patients. Moreover, fiber reduction in the left cingulum (hippocampus) region was negatively associated with speech repetition performance.

Importantly, all patients exhibited an individual pattern of disconnection, suggesting that the anatomy of poststroke fiber abnormalities does not follow a common general outline, but it is individually variable and may be dependent upon the location of the necrotic tissue and hypoperfusion states during the acute stroke. Given the individual variability of disconnection and the anatomical relationship with the necrotic lesion, it is also unlikely that normal physiological asymmetry prestroke may explain these findings. Nonetheless, it is not possible to determine if the observed asymmetry is exclusively the result of fiber loss within the damaged hemisphere or the consequence of compensatory increase in fibers in the contralateral hemisphere.

The relative decrement in connectivity after a stroke is possibly the anatomical substrate for the many disconnection syndromes, whereby a nonlesional cortical area is rendered dysfunctional after a stroke, albeit being spared by the necrotic ischemic lesion.

The technique employed in this study may enable the direct anatomical mapping of structural diaschisis, which represents the sudden loss of function from a region spared by the stroke but directly linked to the necrotic area. For example, diaschisis is particularly important in the context of aphasia, where parietal [19] and cerebellar [20] and subcortical lesions may lead to functional impairment of cortical language areas [4, 6, 21].

Diaschisis has been demonstrated to exist after a remote lesion, and regression of diaschisis is postulated to play a significant part in recovery from language deficits after the stroke [22]. For this reason, a more modern outlook in the investigation of behavioral deficits and recovery after stroke should take into account the remote consequences of damage [23]. A connectivity-based approach may fully disclose the extent of neurological impairment in relation to, for example, aphasia and neglect [24]. In this context, the methodology presented in this paper can be used to map the extent of brain injury beyond the visible necrosis and provide an extension to classic voxel lesion symptom mapping [25]. A connectivity-based approach has the potential to greatly expand the understanding of the relationship between symptoms and structural abnormalities.

## Figures and Tables

**Table 1:** Demographic, behavioral, and anatomical data for patients included in this study.

Subject	*P*1	*P*2	*P*3	*P*4	*P*5	*P*6	*P*7	*P*8
Demographic information								
Age (years)	50.18	59.91	54.7	58.27	55.13	47.64	53.26	37.21
Gender	M	M	F	M	M	F	M	F
Race	C	C	C	C	C	C	C	C
MRI time after stroke (months)	12	61	62	17	50	102	30	68

Behavioral performance								
WAB AQ	72.5	50.9	57.1	51.5	54.1	66.9	46.9	33.2
WAB speech content	8	6	7	7	6	7	3	5
WAB fluency score	4	3	4	7	4	4	2	5
WAB comprehension	8.15	9.05	8.25	7.45	6.55	7.05	8.25	5.55
WAB repetition	7.6	1.7	3	2.3	7	7.3	5	0.5
Boston naming test	54	28	19	2	8	53	18	1

Average percentage of voxel-wise fiber reduction in major white matter pathways in the left hemisphere (×100)								
Anterior thalamic radiation	0.016	0.036	0.022	0.004	0.014	0.015	0.020	0.028
Corticospinal tract	0.031	0.128	0.028	0.011	0.001	0.024	0.036	0.011
Cingulum (cingulate gyrus)	0.003	0.006	0.019	0.057	0.018	0.001	0.007	0.010
Cingulum (hippocampus)	0.002	0.006	0.017	0.010	0.036	0.023	0.016	0.055
Forceps major	0.009	0.008	0.035	0.002	0.007	0.011	0.005	0.026
Inferior frontal-occipital fasciculus	0.042	0.056	0.072	0.004	0.049	0.018	0.056	0.069
Inferior longitudinal fasciculus	0.029	0.029	0.025	0.002	0.071	0.024	0.009	0.047
Superior longitudinal fasciculus	0.055	0.036	0.019	0.028	0.069	0.094	0.032	0.031
Uncinate fasciculus	0.034	0.064	0.155	0.003	0.064	0.085	0.106	0.091
Superior longitudinal fasciculus (temporal part)	0.037	0.042	0.016	0.023	0.095	0.204	0.019	0.001

WAB: Western Aphasia Battery; C: Caucasian.
